# The Clinical Efficacy of Autologous Platelet-Rich Plasma Interventional Circulatory Perfusion Combined with Radiofrequency Ablation and Thermocoagulation in the Treatment of Discogenic Low Back Pain

**DOI:** 10.1155/2023/1489905

**Published:** 2023-07-18

**Authors:** Zhong-Liu Cao, Hu Xu, Jia-Qiang Wu, Jiang-Hua Dai, Si-Jian Lin, Ling-Feng Zou, Ling Yu, Hui-Chun Yang

**Affiliations:** ^1^Department of Rehabilitation Medicine, The Second Affiliated Hospital of Nanchang University, Nanchang 330000, China; ^2^Department of Blood Transfusion, The Second Affiliated Hospital of Nanchang University, Nanchang 330000, China

## Abstract

**Objective:**

In this study, we aimed to explore the efficacy of the autologous platelet-rich plasma (PRP) interventional circulatory perfusion combined with radiofrequency ablation and thermocoagulation (RFAT) in the treatment of discogenic low back pain (DLBP).

**Methods:**

From January 2020 to November 2022, 158 patients of the Second Affiliated Hospital of Nanchang University were selected as the study subjects, and 24 patients met the exclusion criteria. The 134 patients who met the inclusion criteria were divided into 65 patients in the control group (3 patients lost to follow-up) and 69 patients in the observation group (5 patients lost to follow-up), so 126 patients were actually completed the study, including 62 patients in the control group and 64 patients in the observation group. The control group responsible disc received RFAT, and an interventional circulatory perfusion was performed; the observation group received RFAT, and an interventional circulatory perfusion was performed, and then autologous PRP 2 ml was injected. Visual Analog Scale (VAS) and Oswestry Disability Index (ODI) were performed before and 4 and 8 weeks after treatment, and the efficacy was evaluated at 4 and 8 weeks after treatment. The changes of lumbar disc MRI before and after treatment were observed.

**Results:**

The differences in the Visual Analog Scale (VAS) scores and the Oswestry Disability Index (ODI) between the observation group and the control group before the treatment were not statistically significant (*P* > 0.05 in both). However, four weeks and eight weeks after the treatment, the VAS scores and the ODIs were significantly lower in both groups than those before the treatment (*P* < 0.05 in both). In terms of the therapeutic efficacy, eight weeks after the treatment, the total effective rates in the control group and the observation group were 67.7% and 87.5%, respectively, with the observation group being superior to the control group (*P* < 0.05).

**Conclusion:**

After RFAT, interventional circulatory perfusion combined with autologous PRP intramedullary injection in the lumbar disc is a safe and effective treatment for DLBP, and it had superior long-term effects in improving the clinical symptoms and patient dysfunction than the RFAT and interventional circulatory perfusion.

## 1. Introduction

Discogenic low back pain (DLBP), also known as low back pain (LBP), is a highly common clinical syndrome caused by the intervertebral disc degeneration (IVDD) and can have a severe effect on people's work and daily lives. In the United States, 84% of the population is affected by DLBP to some degree during their lives, and the annual cost of treating LBP is as high as US$500 billion. Its incidence has been increasing year by year, making it currently one of the main causes of physical disability in the middle aged and the elderly [1–3].

At present, DLBP is mostly treated with radiofrequency thermocoagulation (RFT). Percutaneous RFT of the intervertebral disc can directly degenerate, coagulate, and reduce the physical volume of the nucleus pulposus (NP) of the protruding diseased part and relieve the pressure, while rarely damaging the normal NP tissue. Moreover, the thermal coagulation of the annulus fibrosus (AF) can successfully block the pain signal transmission of its sinus vertebral nerve (SVN) endings [4, 5]. In addition, RFT has the electrothermal coagulation effect of an electric knife, which means it can coagulate and repair the AF of the damaged intervertebral disc [6]. However, it has no regenerative repair effect on the NP, and since IVDD gradually increases with age, the long-term effect of RFT is poor, and most patients are prone to a recurrence of DLBP [7].

In recent years, the rise of autologous biotechnology, such as the stem cell transplantation and platelet-rich plasma (PRP) injection therapy, has brought new hope for a more effective treatment of degenerative diseases in the elderly population [8–10], and PRP is now widely used in clinical practice. Research has found that PRP is also particularly suitable for treating DLBP caused by IVDD for several reasons [11]. First, PRP contains a much higher concentration of platelets than the physiological concentration. After activation, numerous growth factors, such as transforming growth factor-*β*1, platelet-derived growth factor, insulin-like growth factor, and epidermal growth factor, are released by the platelets to mediate tissue repair. Second, PRP contains various anti-inflammatory factors, including interleukin-receptor antagonist, interleukin 4 (IL-4), IL-10, and IL-13. Finally, the adhesion factors and chemokines in PRP may recruit endogenous stem cells to the NP and promote the regeneration of NP cells and collagen secretion, together with the regeneration and repair of the degenerated intervertebral disc [12–15]. However, few clinical investigations have been reported concerning the use of PRP for DLBP. In the present study, the clinical efficacy of an autologous PRP perfusion via an interventional microcirculation system (IMCS), as developed by Dai et al. [16, 17], combined with RFT, was observed in patients with DLBP.

## 2. Materials and Methods

### 2.1. General Materials

With the approval of the Ethics Committee of the Second Affiliated Hospital of Nanchang University, patients with DLBP attending the hospital between January 2020 and November 2022 were selected as the study objects. All the patients underwent a magnetic resonance image (MRI) of the lumbar spine. The NP structure, signal intensity, boundary between the NP and the AF, and disc height were evaluated according to the Pfirrmann grading system.

The inclusion criteria were as follows: (1) patients with recurrent or persistent pain in one side or both sides of the back for more than three months and a single-segment degenerative disc herniation of grade I (with no rupture of the AF and no herniation of the NP) on the MRI. For the suspicious cases, further discography was performed to confirm the diagnosis; (2) patients aged 30–75 years and of either gender; and (3) patients who gave informed consent.

The exclusion criteria were as follows: (1) patients with a lumbar disc herniation of greater than grade I; (2) patients who had scoliosis or spinal fractures or had undergone spinal surgery; (3) patients with severe osteoporosis; (4) patients with lumbar tuberculosis, infections, tumors, or other diseases; (5) patients with trunk neuromuscular diseases; (6) pregnant or lactating women; (7) patients with rheumatism, rheumatoid arthritis, or systemic lupus erythematosus; or (8) patients with mental illness or severe cognitive dysfunction; (9) patients complicated with necrosis of the femoral head; and (10) patients complicated with lumbar spondylolisthesis or spinal canal stenosis.

**Figure 1:**
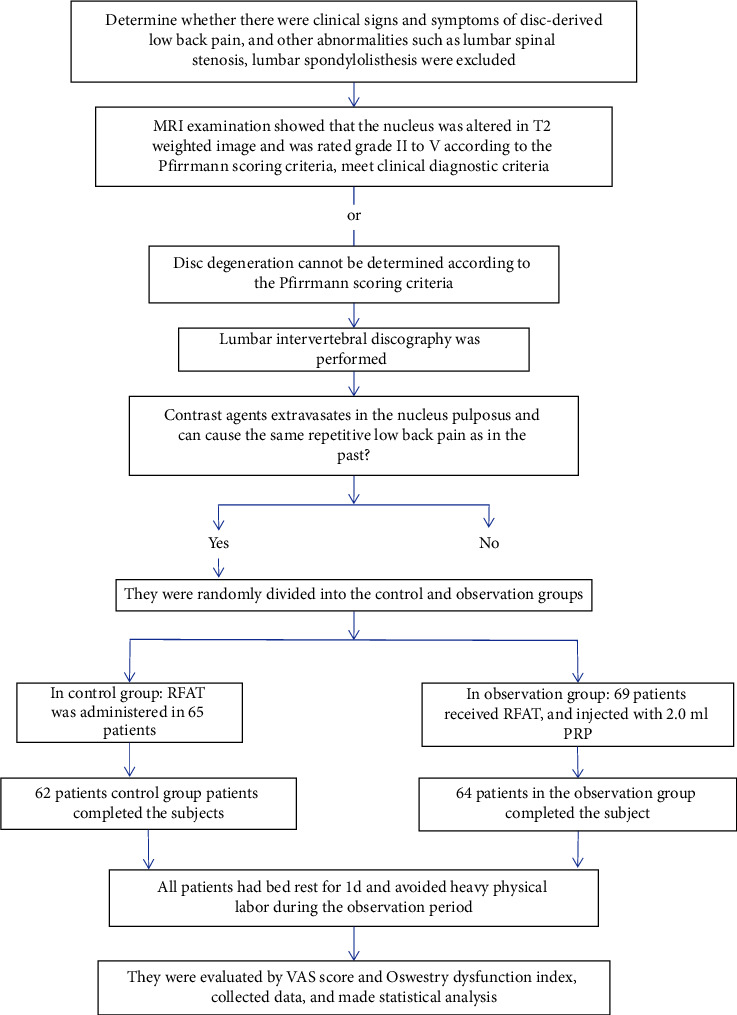
A flowchart including the total, included, excluded, and withdrawn patient numbers.

A flow chart including the total, included, excluded, and withdrawn patient numbers is illustrated in Figure 1. Finally, a total of 126 patients were included, and they were randomly divided into a control group (*n* = 62) and an observation group (*n* = 64). In the control group: 38 males and 24 females; age 32 to 68 years, mean (49.32 ± 6.42); disease duration 6 to 13 months, mean (9.47 ± 2.36) months. In the observation group: 34 males and 30 females; age 32 to 73 years, mean (49.84 ± 5.67); duration 4 to 16 months, mean duration (10.45 ± 4.32) months. No difference was found in the baseline data between the two groups (*P* > 0.05); the data are displayed in Table 1.

This study was approved by the Ethics Committee of the Second Affiliated Hospital of Nanchang University (approval number: (2019) No. (105)). In addition, all patients signed the informed consent by themselves.

### 2.2. Surgical Methods

The control group received targeted RFAT, while the observation group received targeted RFAT combined with PRP injection and interventional circulatory perfusion.

Before the PRP procedure, the physical condition of the recipient was assessed by a transfusion physician to ensure safe collection. After the assessment, the patient's elbow vein access was established, and the preparation of PRP was performed as follows: platelet monocollection was done by the requirements of the “Blood Station Quality Management Standards” and “Quality Requirements for Whole Blood and Component Blood.” The autologous platelets were collected for autologous treatment, and the rest of the components were returned for transfusion by following the laws and regulations related to autologous blood transfusion. The Nangal Automatic Blood Component Separator carried out the platelet-rich plasma preparation (Model: NGL XCF3000 (Sichuan Nangal Biomedical Co., Ltd.)) (the concentration of platelet is 2.0∼3.0 × 10^11^/250 mL bag, i.e., 800∼1000 × 10^9^/L, and the average use is 2.81 × 10^9^/L) for patients with slightly lower platelets. Platelets can also reach more than 500 × 10^9^/L, which can be adjusted according to each patient's PRP collection before the routine blood test indicators, the required collection volume, and the corresponding concentration to achieve the requirements, the collection of samples left for testing, in line with the needs of the PRP treatment concentration (the universal scholarly research pointed out that platelet concentration of 500–1000 × 10^9^/L to promote the best effect of repair). Fresh platelets were put into the platelet shaker (Model: SJW-IB (Shandong Sanjiang Medical Technology Co., Ltd.)), 20–24°C shaking and stored for five days effectively, and the rest were put into the ultralow-temperature refrigerator (Model: MDF-382 (Panasonic Cold Chain (Dalian) Co., Ltd.)) −80°C for storage and thawed using a 37°C water bath (Model: JXH-II (Guangzhou Jinxiangying)) to rewarm Thaw for not less than 5 minutes. After collection, the patient was asked to lie down and could eat to replenish energy.

**Figure 2:**
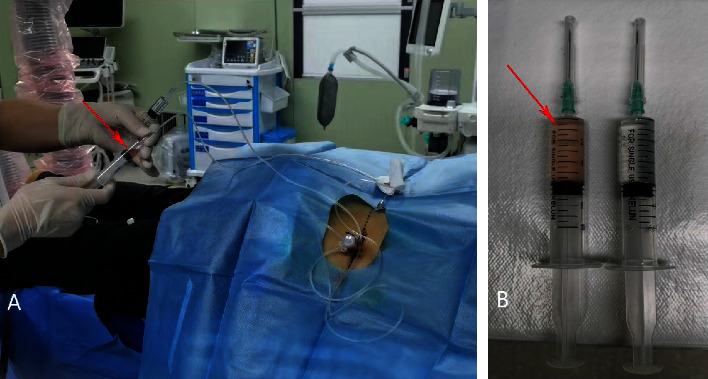
(a) After RFAT in both groups, the accumulated cautery products and a large amount of acidic metabolic waste solution were gradually washed out by circulating perfusion (indicated by red arrows), and then the observation group was injected with 2.0 ml PRP. (b) Yellow-red cloudy liquid in the syringe shown by the red arrow (potential mixture of small red blood cells, coagulated fibrin, and burning necrotic tissue. The right-position syringe is clear saline that acts as a contrast). Principles of interventional microcirculation perfusion therapy: on the one hand, decompression of diseased intervertebral disc. Eliminating inflammatory products, burning necrosis, and metabolic waste to improve the adverse effects of the damaged microenvironment on the formation of proteoglycans of the nucleus pulposus cells; on the other hand, the input of the autologous PRP. The coagulation mechanism is activated in the minimally invasive environment. Prompted the PRP gel formation. Progressive release of multiple bioactive factors. While receiving authentic stress stimulation in situ. The recruitment of mesenchymal stem cells homing under the stimulation of three-dimensional networked biological signals such as biomechanics. Colonization differentiated into highly active disc cells. Involved in the synthesis of aggrecan and collagen. Promote water absorption and hydration of the disc. Simultaneously, regeneration and repair of collagen fiber rings. Not only delay degenerative manifestations such as disc dehydration. Also, reconstructed the regenerative microenvironment of the intervertebral disc.

After entering the operating room, life indications such as venous passage, oxygen inhalation, blood pressure, and heart rate monitoring were established. (1) Targeted RFAT: patients in both the control and observation groups were made to lie in the prone position, with an abdominal pillow. The puncture point was positioned and marked under a C-arm X-ray machine. After the routine towel laying disinfection, the puncture was performed under an X-ray guidance and local anesthesia. In case of a breakthrough and the tip of the needle reached the middle posterior third of the diseased disc close to the midline of the spine within the nucleus pulposus adjacent to the annulus fibri, the sensory and motor nerve stimulations were performed. Then, the targeted RFAT was conducted; (2) interventional circulatory perfusion operation: after RFAT was conducted by the use of IMCS, interventional circulatory perfusion was performed in both the control and observation groups. Firstly, the radiofrequency electrode needle was subsequently extracted, but the outer tube of the puncture needle was left still in the original position. Secondly, an interventional microcirculation perfusion casing system (a multifunctional stem cell transplant gun, patented: ZL201921035597.8 and IMCS has been granted invention patent authorization, ZL202110827937.6) was inserted into the outer tube of the puncture needle and under fluoroscopy ensured that the IMCS tip was accurately located at the therapeutic target. Thirdly, the interventional circulatory perfusion was performed and the burning products and the acidic metabolic fluid accumulated in the degenerative disc were slowly washed out; (3) after the abovementioned two steps, a 2.0 ml PRP was injected into the target site in the observation group. (Figure 2(a)). Then, the IMCS was pulled out along with the coat needle. A sterile dressing was applied externally to the puncture point. All patients had bed rest for 24 h, avoided lifting weights, and avoided excessive fatigue and heavy physical labor during the observation period.

Yellow-red cloudy liquid in the syringe is shown by the red arrow (potential mixture of small red blood cells, coagulated fibrin, and burning necrotic tissue. The right-position syringe is clear saline that acts as a contrast (Figure 2(b)).

### 2.3. Therapeutic Efficacy Assessment

Complications during the PRP injection were observed. The pain levels were assessed using the Visual Analog Scale (VAS), and the functional impairment level was assessed using the Oswestry Disability Index (ODI) before treatment and at four and eight weeks after treatment. The therapeutic efficacy was classified into four grades [18] as follows: (1) “cured” meant there were no clinical symptoms, and the lumbar function was normal; (2) “very effective” meant all the symptoms, or at least the main ones, had disappeared, and there was a recovery of the lumbar function allowing the person to participate in normal labor or work; (3) “effective” meant the main symptoms had disappeared, and the improvement in the lumbar function was such that the person could now take care of themselves or participate in labor and work; and (4) “ineffective” meant there was little or no improvement. The total effective rate was calculated as the number of cases graded as (cured + very effective + effective) ÷ the total number of cases × 100%. The T2-weighted image (T2WI) signal changes of the intervertebral discs were observed using the magnetic resonance imaging (MRI) before and after treatment to evaluate the effects of hydration enhancement on the degenerated discs in the observation and control group.

### 2.4. Statistical Methods

The VAS and ODI data of 126 patients were recorded in Microsoft Excel and analyzed using SPSS™ Statistics v22.0 statistical software. The measurement data were expressed as means ± standard deviations (*x* ± *s*) using a *t*-test or a repeated-measures analysis of variance. The countable data were expressed in rates or percentages using *χ*^2^ test. A value of *P* < 0.05 was considered statistically significant.

## 3. Results

### 3.1. Comparison of Therapeutic Efficacy

The total effective rate of the control group was 67.7% and that of the observation group was 87.5% (Table 2). The efficacy of the observation group was significantly higher than that of the control group (*χ*^2^ = 4.05, *P* < 0.05).

### 3.2. Comparison of the Visual Analog Scale Scores and the Oswestry Disability Indexes

**Figure 3:**
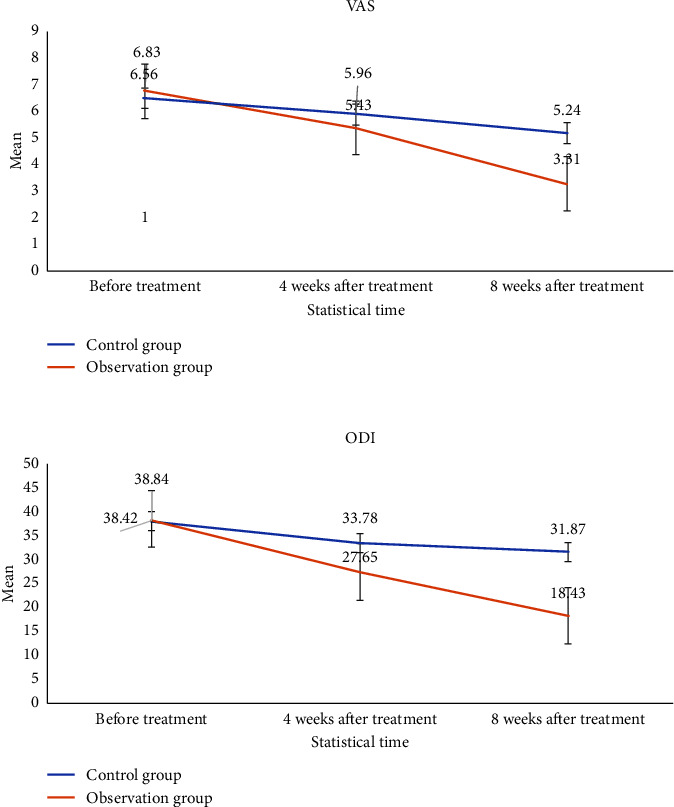
Changes in the visual analog scale scores and Oswestry disability index before and after treatment in the two groups.

Before treatment, the differences in the VAS scores and the ODIs were not statistically significant between the two groups (*P* > 0.05 in both). At four and eight weeks after treatment, the VAS scores and ODIs in the two groups were significantly lower than those before treatment (*P* < 0.05). However, the differences in the VAS scores and ODIs between the observation group and the control group at four weeks after treatment were not statistically significant (*P* > 0.05). At eight weeks after treatment, the VAS scores and ODIs in the observation group were significantly lower than those in the control group (*P* < 0.05). With regard to the intragroup comparison, in the observation group, the reductions between four weeks and eight weeks after treatment were not noteworthy (*P* > 0.05), and the differences were not statistically significant. However, in the observation group, the reductions in the VAS scores and ODIs between four weeks and eight weeks after treatment were marked (*P* < 0.05), and the differences were statistically significant (Table 3). Notably, the reduction in the VAS scores and ODIs in the observation group continued until eight weeks postoperatively, whereas the rate of reduction in the VAS scores and ODIs in the control group was not obvious from four to eight weeks postoperatively (Figure 3). These results suggest that both RFAT and RFAT combined with autologous PRP perfusion via an IMCS can be effective in the treatment of DLBP caused by IVDD, but the latter might be more effective than RFAT in the long run (at least up to eight weeks later).

In the observation group, there were no serious complications during the PRP perfusion, such as nerve root injury, multifidus abscess, cauda equina syndrome, spondylitis, or thrombosis, and only a few patients had mild-to-moderate swelling and pain, which disappeared within 1-2 days.

### 3.3. Changes in the Magnetic Resonance Images of Lumbar Discs in Both the Control and Observation Groups

**Figure 4:**
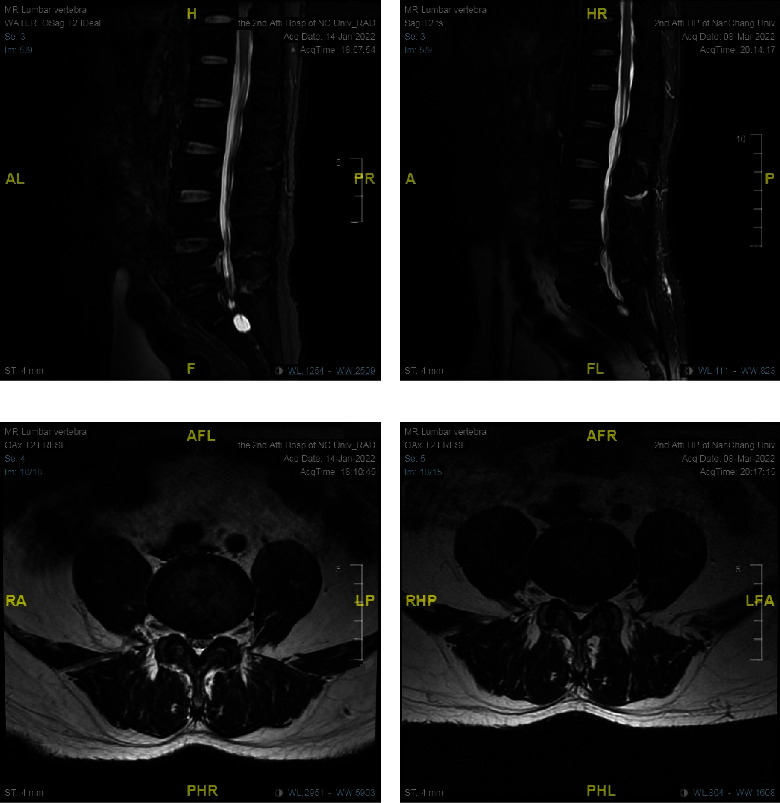
(a) T2-weighted image of MRI in the control group in 2022-1-14; (b) MRI T2-weighted image in 2022-3-8; the height and penetration of L4-L5 disc showed no improvement than before treatment, and the sign of disc “bulging” still exists; (c) MRI performance of L4-L5 transverse section in the control group before treatment in 2022-1-14; (d) MRI performance of L4-L5 transverse section after 2 months of 2022-3-8 treatment.

**Figure 5:**
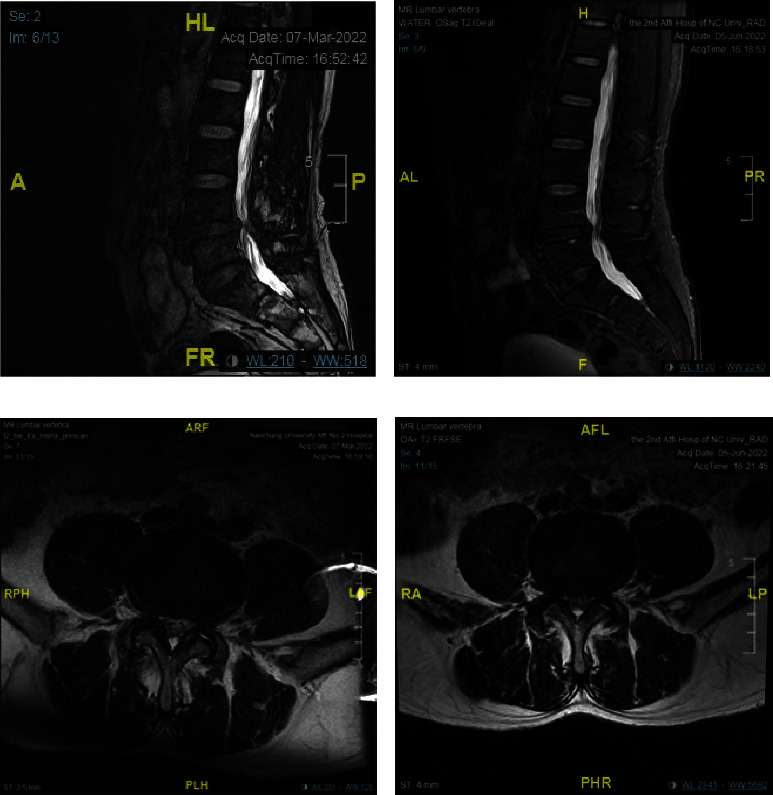
(a) T2-weighted image of MRI before treatment in 2022-03-07 observation group; (b) T2-weighted image of MRI in 2022 after 2 months of treatment; the height and thickness of L4-L5 disc increased slightly compared with before treatment, and the degree of “bulging” of the diseased disc in (b) was reduced compared with (a); (c) T2-weighted image performance of L4-L5 disc level before treatment in 2022-03-07 observation group; (d) T2-weighted image performance of L4-L5 disc level 2 months after treatment in 2022-06-05 observation group, indicating that the MRI T2WI signal of the same disc was increased compared with preoperative, indicating the enhanced hydration of disc after treatment and no obvious fat infiltration.

The magnetic resonance images (MRIs) of typical patients in the control and observation groups are shown in Figures 4 and 5, respectively. In the observation group, the MRI of the lumbar discs before treatment suggested that the T2WI signals of L4-L5 discs were reduced in both the sagittal and cross-sectional views. The T2WI signals in the MRI of the L4-L5 discs in the right panel were significantly higher than those of the same segment in the left panel after the PRP perfusion treatment via IMCS. However, in the control group, no increase was observed in the T2WI signal of MRI.

## 4. Discussion

The lumbar spine is the section that bears the most weight, has the greatest range of motion, and plays the most prominent role. IVDD is known for the underlying cause of DLBP [19], and it often leads to the following: (1) mechanical changes: physiologically, the adult intervertebral disc is almost devoid of blood vessels, and only small vessels from the segmental arterial branches penetrate around the AF, mostly at the anterior and posterior edges of the disc. These innate anatomical weaknesses cause the lumbar intervertebral discs to undergo extreme biomechanical stimulation, and over time, the stress leads to the less elastic NP crossing the weakened AF [20]; (2) pain changes: the level of pain depends on the pain-sensitive tissues of the spinal canal—AF of the disc, the posterior longitudinal ligament, and the SVN endings distributed on the surface of the dura [21]; and (3) chemical changes: the locally injured intervertebral disc transforms from being self-contained to contact with the outside world and due to the antigenic properties of intervertebral disc tissues, an immune-inflammatory response is triggered, resulting in an autoimmune reaction in the disc and chronic inflammation and LBP [22].

The targeted RFT therapy is a common method for the treatment of DLBP due to IVDD [23]. This therapy involves the emission of a high-frequency RF current through a magnetic field. The RF current generates a change in the magnetic field at the tip of a working electrode, which causes molecular movement and heat generation in the target tissue covered by the magnetic field, and the thermal coagulation destroys the tissue in the target area. It can denature, coagulate, and reduce the physical volume of the protruding diseased part of the NP, thereby relieving the pressure. As it rarely injures the normal NP tissue, it is a considerable improvement on the traditional method of treatment, which involves destroying the normal NP. The AF-targeted thermocoagulation can successfully block the pain signaling of the SVN endings within the AF, while the RFT has the electrothermal coagulation effect of an electric knife and can restore the AF of the damaged disc. Although the targeted thermal coagulation alone is effective, most patients relapse within 1–3 years, so it provides a purely temporary physical relief that does not reverse the pathological process and, therefore, has less than ideal long-term results [24].

No matter whether the pain is caused by the mechanical or chemical changes, it is essentially due to a disruption of the survival microenvironment of the NP cells. The level of pain may be determined physiologically by detecting the osmotic pressure and pH of the injured area [25, 26]. Just as changes in osmotic pressure, pH, or chemical composition indicate pain in other organs, for instance, toothache or stomachache, the same is true for discogenic pain.

Nachemson [27] measured the pH of prolapsed discs in vivo with the use of a ladder electrode needle, and when the pH was <7, an inflammatory response was observed in the nerve endings. However, the same reaction did not always occur in alkaline pH, and in pH ≤6.1, the nerve roots were found to be encapsulated in thick active scar tissue. They believed that the chemical composition of the prolapsed disc tissue might also contribute to the symptoms and was independent of its size. Even if the disc was not prolapsed, the nerve structures surrounding the disc, such as the posterior longitudinal ligament, could be irritated by the chemical changes occurring within the structure. As for other organs, a weakened metabolism was believed to induce the chemical processes that altered the osmotic pressure and pH of the disc tissue. They also hypothesized that protein hydrolases in the lysosomes of the connective tissue might act as catalysts, prompting the release of acidic amino acids from the proteoglycan complexes of the disc matrix, which might lead to a decrease in pH, and that an increased concentration of free acidic mucopolysaccharides might further decrease the pH. In addition, Diamant et al. [28] reported that the disc pH measured intraoperatively was inversely proportional to the concentration of the lactate and a lower pH was correlated with a higher lactate concentration and vice versa. They speculated that, if the microenvironment within the disc continued to deteriorate with the maintenance of a relatively high hydrostatic pressure, easily soluble acidic metabolites might be extruded from the disc under high pressure, triggering an inflammatory response in the peripheral nerve fibers. Meanwhile, this would induce changes in the proteoglycan and collagen and accelerate IVDD. It was also suggested that PRP could accelerate IVDD by promoting the NP cells to synthesize aggrecan and collagen and by directly stimulating the differentiation of bone marrow stromal cells into mature intervertebral disc cells, these cells being the two main components of the intervertebral disc extracellular matrix. Moreover, it was thought that, as the main proteoglycan, aggrecan promotes water absorption and disc hydration, thereby delaying the degenerative manifestations such as disc dehydration [29].

It was expected that DLBP caused by IVDD could be treated effectively by improving the local inflammatory metabolic microenvironment of the degenerated disc and promoting the synthesis of aggrecan and collagen. In the present study, an IMCS annular perfusion cannula was used to puncture the same target of the intervertebral disc with RFT under the guidance of a C-arm X-ray machine. Dai Jianghua et al. independently developed IMCS, through the cyclic perfusion effect of normal saline, the yellow-red cloudy acidic metabolic fluid accumulated locally in the degenerated intervertebral disc was slowly flushed out (Figure 2), and then 2.0 ml of PRP was injected. The MRI of the degenerated intervertebral discs suggested that the T2WI signal was reduced in both the sagittal and cross-sectional views. It was observed that, with the combined application of PRP, the T2WI signal of the degenerated disc in the same segment was higher in the observation group after treatment than that before surgery (Figures 4 and 5), suggesting that PRP promoted the hydration of the degenerated disc.

Under normal physiological conditions, the degeneration of the lumbar disc in humans as they age is accompanied by changes in the NP cells and the extracellular matrix, that is, a deterioration of the intradisc microenvironment, which eventually leads to the structural destruction of the disc [30]. Current clinical investigations and animal experiments have demonstrated the presence of cellular necrosis within the degenerated disc, that is, activation of the apoptotic pathways due to nutrient deficiency within the disc, causing cellular self-regulation, which leads to disc degeneration [31–33]. Given the unique advantages of both RFT and autologous PRP regenerative repair, the two therapies were combined to play a coordinating role in promoting each other. It was speculated that the local high temperature generated by RFT might destroy the hypersensitive nerve receptors in the intervertebral disc, reduce the nociceptive peripheral nerves, block the nociceptive nerve conduction pathway, and improve the symptoms of LBP in patients. The acidic metabolic waste accumulated locally in the degenerated disc might be gradually discharged from the body by means of perfusion via IMCS (Figure 2). In the present study, with the completion of the thermal coagulation and perfusion via IMCS, approximately 2 ml of PRP was injected into the NP to stimulate collagen regeneration in the NP and slow down the degeneration process of the lumbar disc. By comparing RFAT combined with PRP perfusion (the observation group) with RFAT (the control group), it was found that, at four weeks after treatment, the pain symptoms were relieved in both the observation group and the control group. However, at eight weeks after treatment, the observation group had further improvement, while the control group had no significant change. Therefore, it is suggested that intramedullary autologous PRP perfusion via IMCS combined with RFAT might be a safer and more effective treatment modality than RFAT, particularly in the long term, and the combined therapy appears likely to fundamentally slow down the degeneration process of the lumbar disc.

Many animal and clinical trials in China and elsewhere have shown the synergistic effect of multiple growth factors improving the symptoms of DLBP by effectively delaying the degeneration of intervertebral discs [34, 35]. It appears that the new method of PRP perfusion via IMCS could provide a beneficial regenerative microenvironment for the regeneration and repair of the diseased tissues and organs. The high concentration of platelets contained in PRP releases multiple growth factors after activation to promote mitosis, proliferation, and differentiation by the synthesis of NP cells and the extracellular matrix. Nevertheless, the mechanism of IVDD is not yet fully understood, and the quality control of PRP still needs to be improved. It is therefore suggested that a multicenter double-blind randomized controlled study with a larger sample size and longer follow-up duration should be conducted in the future. Our next step is to increase the evaluation results of patient's VAS score and ODI after 6 months and 12 months.

Evidently, there are some limitations to this study. First, the sample size of the study was small, and more prospective, large-sample, and multicenter studies should be conducted to validate the findings. Second, the evaluation of the treatment efficacy was mainly based on the clinical scores and MRI; however, the histological observations may be more convincing. Moreover, comparisons between the two groups were performed only at four and eight weeks postoperatively, and longer postoperative observations should be supplemented in the future studies.

## 5. Conclusion and Outlook

In conclusion, it appears that IMCS can drive in vivo microcirculation through extracorporeal circulation, realizing a dual circulation-driven model of internal and external circulation, overturning the traditional paradigm that the microcirculation reconstruction of the diseased tissues and organs in situ can only be achieved via internal circulation. It is hoped that IMCS could be used to loosen and decompress the diseased tissues and perfuse the circulation in an almost noninvasive manner, which could improve the local inflammatory microenvironment of the diseased tissues and organs by discharging the metabolic waste from the body in a timely and convenient manner. Biomarkers, such as metabolomics and proteomics, could be analyzed at the molecular level of the discharged material, and, furthermore, the use of autologous biotechnology could provide a precise and regenerative green treatment plan for the complete cure of degenerated and damaged tissue and organ diseases. The precise and efficient combination of IMCS and autologous biotechnology is promising for clinical application.

## Figures and Tables

**Table 1:** Basic data of two groups of patients (*x* ± *s*).

Group	*n*	Age	Gender (male/female)	Average course of disease
The control group	62	49.32 ± 6.42	38/24	9.47 ± 2.36
The observation group	64	49.84 ± 5.67	34/30	10.45 ± 4.32
*P*		0.964	0.354	0.612

**Table 2:** Comparison of the clinical efficacy at 8 weeks after the treatment between the two groups.

Group	*n*	Cure	Obvious effective	Effective	Ineffective	Total effective/ineffective
The control group	62	3	16	23	20	42/20
The observation group	64	9	20	27	8	56/8
*P*						0.038^*∗*^

^*∗*^There was a significant difference between two groups (*P* < 0.05).

**Table 3:** Comparison of the VAS score and the ODI of the two groups (*x* ± *s*).

	*n*	Indicator	Before treatment	4 weeks after treatment	8 weeks after treatment	*P*1	*P*2	*P*3
The control group	62	The VAS score	6.56 ± 1.32	5.96 ± 1.62	5.24 ± 1.05	0.046^*∗*^	0.034^*∗*^	0.352
The observation group	64		6.83 ± 1.43	5.43 ± 0.67	3. 31 ± 1.76	0.043^*∗*^	0.023^*∗*^	0.035^*∗*^
*P*4			0.832	0.214	0.042^*∗*^			
The control group	62	The ODI (%)	38.42 ± 5.87	33.78 ± 4.82	31. 87 ± 4.73	0.045^*∗*^	0.041^*∗*^	0.276
The observation group	64		38.84 ± 6.03	27.65 ± 8.37	18. 43 ± 4.72	0.039^*∗*^	0.021^*∗*^	0.024^*∗*^
*P*4			0.965	0.167	0.032^*∗*^			

*Note.P*1 indicates the *P* value of the VAS score or ODI at 4 weeks after treatment compared with the VAS score or ODI before treatment; *P*2 indicates the *P* value of the VAS score or ODI at 8 weeks after treatment compared with the VAS score or ODI before treatment; *P*3 indicates the *P* value of the VAS score or ODI at 8 weeks after treatment compared with the VAS score or ODI before treatment; *P*4 indicates the *P* value of VAS score or ODI comparison between two groups at different time points. ^*∗*^indicates a significant difference (*P* < 0.05).

## Data Availability

The data that support the findings of this study are available from the corresponding author upon reasonable request.

## References

[1] Kague E., Turci F., Newman E. (2021). 3D assessment of intervertebral disc degeneration in zebrafish identifies changes in bone density that prime disc disease. *Bone Res*.

[2] Zhao L., Manchikanti L., Kaye A. D., Abd-Elsayed A. (2019). Treatment of discogenic low back pain: current treatment strategies and future options-a literature review. *Current Pain and Headache Reports*.

[3] Yang S., Zhang F., Ma J., Ding W. (2020). Intervertebral disc ageing and degeneration: the antiapoptotic effect of oestrogen. *Ageing Research Reviews*.

[4] Zeng Z., Yan M., Dai Y., Qiu W., Deng S., Gu X. (2016). Percutaneous bipolar radiofrequency thermocoagulation for the treatment of lumbar disc herniation. *Journal of Clinical Neuroscience*.

[5] Barendse G. A., van Den Berg S. G., Kessels A. H., Weber W. E., van Kleef M. (2001). Randomized controlled trial of percutaneous intradiscal radiofrequency thermocoagulation for chronic discogenic back pain: lack of effect from a 90-second 70 C lesion. *Spine*.

[6] Freeman B. J., Mehdian R. (2008). Intradiscal electrothermal therapy, percutaneous discectomy, and nucleoplasty: what is the current evidence?. *Current Pain and Headache Reports*.

[7] Mohanty S., Dahia C. L. (2019). Defects in intervertebral disc and spine during development, degeneration, and pain: new research directions for disc regeneration and therapy. *Wiley interdisciplinary reviews. Developmental biology*.

[8] Barry F., Murphy M. (2013). Mesenchymal stem cells in joint disease and repair. *Nature Reviews Rheumatology*.

[9] Chen L., Deng C., Li J. (2019). 3D printing of a lithium-calcium-silicate crystal bioscaffold with dual bioactivities for osteochondral interface reconstruction. *Biomaterials*.

[10] Tuakli-Wosornu Y. A., Terry A., Boachie-Adjei K. (2016). Lumbar intradiskal platelet-rich plasma (PRP) injections: a prospective, double-blind, randomized controlled study. *PM&amp;R*.

[11] Wu P. I., Diaz R., Borg-Stein J. (2016). Platelet-rich plasma. *Physical Medicine and Rehabilitation Clinics of North America*.

[12] Akeda K., Takegami N., Yamada J. (2022). Platelet-rich plasma-releasate (PRPr) for the treatment of discogenic low back pain patients: long-term follow-up survey. *Medicina*.

[13] Carluccio A., Veronesi M. C., Plenteda D., Mazzatenta A. (2020). Platelet-rich plasma uterine infusion and pregnancy rate in barren mares with chronic degenerative endometritis. *Polish Journal of Veterinary Sciences*.

[14] Raeissadat S. A., Ghazi Hosseini P., Bahrami M. H. (2021). The comparison effects of intra-articular injection of Platelet Rich Plasma (PRP), Plasma Rich in Growth Factor (PRGF), Hyaluronic Acid (HA), and ozone in knee osteoarthritis; a one year randomized clinical trial. *BMC Musculoskeletal Disorders*.

[15] Cook C. S., Smith P. A. (2018). Clinical update: why PRP should Be your First choice for injection therapy in treating osteoarthritis of the knee. *Curr Rev Musculoskelet Med*.

[16] Dai J., Li W., Deng J., Luo J., Dai M., Nie T. (2017). A new strategy for repairing large bone defects using an interventional micro-circulation system. *Allied Academies*.

[17] Deng J.-H., Li W., Dai J. H. (2019). Rapid repair of goat large segmental loading bone defect and functional reconstruction by interventional micro-circulation system. *Journal of Biomaterials and Tissue Engineering*.

[18] Shirado O., Doi T., Akai M., Fujino K., Hoshino Y., Iwaya T. (2007). An outcome measure for Japanese people with chronic low back pain: an introduction and validation study of Japan Low Back Pain Evaluation Questionnaire. *Spine 1976*.

[19] Peng B., Hao J., Hou S. (2006). Possible pathogenesis of painful intervertebral disc degenera tion. *Spine*.

[20] Raj P. P. (2008). Intervertebral disc: anatomy-physiology-pathophysiology-treatment. *Pain Practice*.

[21] Quinones S., Konschake M., Aguilar L. L. (2021). Clinical anatomy of the lumbar sinuvertebral nerve with regard to discogenic low back pain and review of literature. *European Spine Journal*.

[22] Podichetty V. K. (2007). The aging spine: the role of inflammatory mediators in intervertebral disc degeneration. *Cellular and Molecular Biology*.

[23] Zhang D., Zhang Y., Wang Z., Zhang X., Sheng M. (2015). Target radiofrequency combined with collagenase chemonucleolysis in the treatment of lumbar intervertebral disc herniation. *International Journal of Clinical and Experimental Medicine*.

[24] Yao X. G., Zhou Y. G. (2006). Treatment of lumbar intervertebral disc protrusion by radiofrequency thermocoagulation and target ablation. *Pain Clinic Journal*.

[25] Liu Q., Tawackoli W., Pelled G. (2015). Detection of low back pain using pH level-dependent imaging of the intervertebral disc using the ratio of R1*ρ* dispersion and-OH chemical exchange saturation transfer (RROC). *Magnetic Resonance in Medicine*.

[26] Hodson N. W., Patel S., Richardson S. M., Hoyland J. A., Gilbert H. T. J. (2018). Degenerate intervertebral disc-like pH induces a catabolic mechanoresponse in human nucleus pulposus cells. *JOR Spine*.

[27] Nachemson A. (1969). Intradiscal measurements of pH in patients with lumbar rhizopathies. *Acta Orthopaedica Scandinavica*.

[28] Diamant B., Karlsson J., Nachemson A. (1968). Correlation between lactate levels and pH in discs of patients with lumbar rhizopathies. *Experientia*.

[29] Urban J. P., Roberts S. (2003). Degeneration of the intervertebral disc. *Arthritis Research and Therapy*.

[30] Sato M., Yamamoto Y., Sakai D., Mochida J. (2004). *Clinical Calcium*.

[31] Wuertz K., Godburn K., Neidlinger-Wilke C., Urban J., Iatridis J. C. (2008). Behavior of mesenchymal stem cells in the chemical microenvironment of the intervertebral disc. *Spine*.

[32] Huang Y. C., Leung V. Y., Lu W. W., Luk K. D. (2013). The effects of microenvironment in mesenchymal stem cell-based regeneration of intervertebral disc. *The Spine Journal*.

[33] Guerrero J., Häckel S., Croft A. S., Hoppe S., Albers C. E., Gantenbein B. (2021). The nucleus pulposus microenvironment in the intervertebral disc: the fountain of youth?. *European Cells and Materials*.

[34] Nazari L., Salehpour S., Hosseini M. S., Hashemi Moghanjoughi P. (2020). The effects of autologous platelet-rich plasma in repeated implantation failure: a randomized controlled trial. *Human Fertility*.

[35] Levi D., Horn S., Tyszko S., Levin J., Hecht-Leavitt C., Walko E. (2016). Intradiscal platelet-rich plasma injection for chronic discogenic low back pain: preliminary results from a prospective trial. *Pain Medicine*.

